# Development and characterization of the replicon system of Japanese encephalitis live vaccine virus SA14-14-2

**DOI:** 10.1186/1743-422X-10-64

**Published:** 2013-02-26

**Authors:** Shi-Hua Li, Xiao-Feng Li, Hui Zhao, Yong-Qiang Deng, Xue-Dong Yu, Shun-Ya Zhu, Tao Jiang, Qing Ye, E-De Qin, Cheng-Feng Qin

**Affiliations:** 1 Department of Virology, State Key Laboratory of Pathogen and Biosecurity, Beijing Institute of Microbiology and Epidemiology, Beijing, 100071, China

**Keywords:** Japanese encephalitis virus (JEV), SA14-14-2, Replicon

## Abstract

**Background:**

Viral self-replicating sub-genomic replicons represent a powerful tool for studying viral genome replication, antiviral screening and chimeric vaccine development. Many kinds of flavivirus replicons have been developed with broad applications.

**Findings:**

The replicon system of JEV live vaccine strain SA14-14-2 was successfully developed in this study. Two kinds of replicons that express enhanced green fluorescent protein (EGFP) and Renilla luciferase (R.luc) were constructed under the control of SP6 promoter, respectively. Robust EGFP and R.luc signals could be detected in the replicon-transfected BHK-21 cells. Furthermore, the potential effects of selected amino acids in the C-terminal of envelope protein on replication were characterized using the replicon system.

**Conclusions:**

Our results provide a useful platform not only for the study of JEV replication, but also for antiviral screening and chimeric vaccine development.

## 

Japanese encephalitis is now recognized as the leading cause of viral encephalitis in Asian countries, including China, Japan, Korea, the Philippines, Thailand, and India [1,2]. Clinical Japanese encephalitis is a severe disease with a high case fatality rate. World Health Organization (WHO) estimates that approximately 50,000 cases of Japanese encephalitis occur each year, resulting in about 10,000 deaths and 15,000 cases of neurological or psychiatric sequelae [3,4]. Japanese encephalitis virus (JEV) is transmitted in an enzootic cycle between *Culex* species mosquitoes and vertebrates, primarily birds with pigs serving as amplifying hosts. In recent years, JEV has begun to spread to other geographic areas such as Pakistan and Australia [5,6]. The geographic expansion and high fatality rates have drawn increasing attention from the international public health community [7].

Vaccination has been recognized as the most reliable and economic measure for protection against Japanese encephalitis. Currently, three kinds of vaccines are available: inactivated vaccine produced in mouse-brain or cell culture and live attenuated vaccine produced on primary hamster kidney (PHK) cells [8]. The live vaccine (SA14-14-2) was initially licensed in 1989 in mainland China, and now exported to most JEV-endemic countries, including India, Sri Lanka, Nepal, Thailand and South Korea under the recommendation of WHO [9]. Large scale immunizations in more than 300 million children have well demonstrated its excellent safety and efficacy profile. Very recently, a novel chimeric JEV live vaccine based on the genetic background of Yellow fever virus (YFV) 17D strain was licensed in Australia and is under active consideration for license in Thailand [10].

JEV belongs to the *Flavivirus* genus in the family *Flaviviridae* together with YFV, dengue virus (DENV), West Nile virus (WNV), Murray Valley encephalitis virus (MVEV) and tick-borne encephalitis virus (TBEV). The genome of JEV is a positive-sense single-stranded RNA molecule comprising 10, 976 nucleotides with a long open reading frame coding for three structural (C, prM, and E) and seven nonstructural (NS1, NS2A, NS2B, NS3, NS4A, NS4B, NS5) proteins. The RNA genome has a type I cap structure at its 5^′^-end and lacks the poly (A) tail at its 3^′^-end [11].

A viral replicon is a self-replicating sub-genomic viral RNA originated from viral genome, which contains viral non-structural genes that are critical for viral genome replication with structural proteins deleted or replaced by foreign genes. This non-infectious replicon provides a valuable platform to study the function and structure of viral genome RNA, express foreign proteins and develop novel vaccines. In recent years, many flavivirus replicons have been developed, including Kunjin virus [12], Tick-borne encephalitis virus [13], DENV [14-17], Yellow fever virus [18,19], and West Nile virus [20-23].

The reverse genetic system of JEV is greatly hampered due to the toxicity of JEV cDNA in bacteria. Despite extensive efforts for many years [24-27], a genetically stable full-length infectious cDNA clone of JEV was not obtained until the bacterial artificial chromosome (BAC) was used as a vector in 2003. Then, several JEV replicons using the BAC vector were constructed based on a Korean JEV strain K87P39 to express the foreign proteins [11]. In this work, we described the preption of a sub-genomic replicon derived from JEV attenuated strain SA14-14-2, as well as, a series of replicons with Enhanced green fluorescent protein (EGFP) and Renilla luciferase (R.luc) reporter genes were constructed and characterized, respectively. These replicons should be useful for studying many aspects of JEV replication, expressing foreign proteins and developing new vaccines.

The low-copy pACNR vector and *E. coli* MC1061 were employed in our experiments [19,20,28]. All the primers used in this study are listed in Additional file 1: Table S1. Firstly, new restriction enzyme sites (*Xho*I, *Bsp*EI, and *Asc*I) were introduced into the multiple cloning site of pACNR by fusion PCR with *Not* I( + )and linker-1(-), resulting in a new vector named pANCR-L1. The 5^′^-half (nucleotides [nt] 1 to 3446), 3^′^-1 (3099 to 7299) and 3^′^-2 (7299 to 10976) fragments covering the full-length JEV SA14-14-2 genomic cDNA were amplified by RT-PCR using high-fidelity M-MLV reverse transcription polymerase (TaKaRa), respectively. Primer pair of F-*Asc*I (+) and J-*Bsp*EI (−) were used to amplify the 5^′^-half fragments encompassing the SP6 promoter. The cDNA product was digested with *Asc*I and *Bsp*EI, and then inserted into the *Asc*I and *Bsp*EI sites of pACNR-L1. Following transformation in competent *E. coli* MC1061 (Invitrogen), positive clones were selected and confirmed by DNA sequencing and named as pANCR-JEV-5. The 3^′^-1 and 3^′^-2 fragments were obtained using the primer pairs of pBRJEV-7289(+), pBRJEV-7289(−) and pBRJEV-7289-3U (+), pBRJEV-7289-3U (−), respectively. Both fragments were first subcloned into pGEM-T-Easy vector (TaKaRa), yielding pT-3099-10976. The pT-3099-10976 was then digested with *Bsp*EI and *Xho*I, and then inserted into the pACNR-L1 to obtain the subclone coding the 3^′^-half fragment and named pANCR-JEV-3. Next, the JR-5-F (1 to 477) and JR-5-S (2468 to 2649) fragments were amplified by RT-PCR using primer sets F-*Asc*I, R-J-Rep and F-J-Rep, R-J-*Bgl*II (introduced another *K*asI site by a silent A to C substitution on position 474 of C gene), and integrated together by overlapping PCR to generate plasmid J-R-5 (ΔprM/E). The J-R-5 (ΔprM/E) fragment was finally digested with *Not*I and *Xho*I, and then ligated into the plasmid pANCR-JEV-3, resulting into the desired JEV replicon pJE3Rep. The constructed JEV replicon pJE3Rep lacks the structural protein coding regions except for the whole C protein and the last 3 amino acids of the E protein (Figure 1). It has been demonstrated that the coding sequence of C protein was important in the RNA cyclization and essential for the replication [29-31]. On the other hand, the last three amino acids of the E protein were retained to ensure proper translocation of the NS1 protein into the lumen of the endoplasmic reticulum [12].

**Figure 1:**
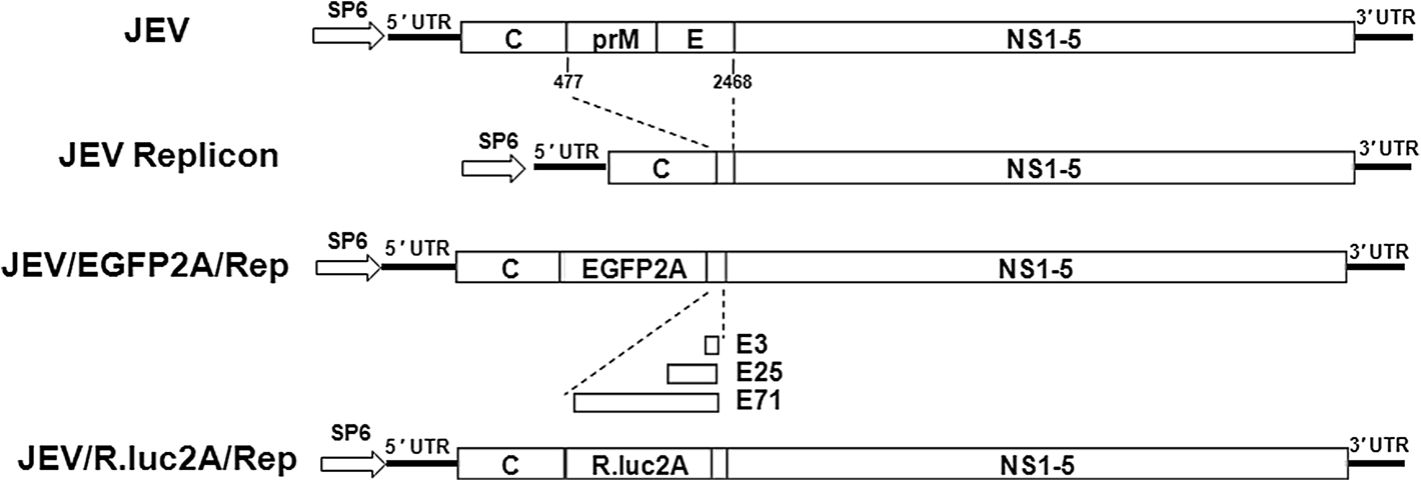
**The schematic representation of JEV replicons constructed in this study.** The prM/E coding region was deleted from the genome of JEV (SA14-14-2) except for the C-terminal amino acid residues of E protein. FMDV-2A was fused downstream of the EGFP and R.luc reporter genes to ensure cytosolic cleavage of heterologous genes.

To assess whether the replicon was functional, the plasmid pJE3Rep was linearized by digestion with the *Xho*I restriction enzyme and then transcribed *in vitro* by using the RiboMax™Large Scale RNA Production system-sp6 Transcription Kit (Promega) in the presence of the m^7^G (5^′^) ppp (5^′^)G cap analogs (Promega). The *in vitro* synthesized RNAs were transfected into BHK-21 cells with Lipofectamine 2000 (Invitrogen) as previously described [32]. Viral RNAs extracted from JEV infected BHK-21 cells were transfected in the same way and used as a positive control. PBS-transfected cells were used as a negative control (Mock). To make sure whether viral protein were successfully expressed by the replicon, the transfected cells were fixed and analyzed by indirect immunofluorescence assay at 72 h post transfection using monoclonal antibodies against E and NS1 proteins of JEV, respectively. As shown in Figure 2A, the negative control did not react with any of the antibodies, and both NS1 and E proteins were detected in the viral RNAs transfected cells. The replicon-transfected cells only reacted with anti-NS1 monoclonal antibody, and no E protein expression was noticed, indicating the JEV replicon could express viral nonstructural protein. Additionally, RT-PCR was carried out for the BHK-21 cells using primers pair F2/R2 and F6/R6 targeting at the prM/E and the NS3 genes. As expected, both genes were detected in viral RNAs transfected cells (lane 3, 4), while only the NS3 gene (lane 2) was detected in the JEV replicon transfected cells. No PCR products were amplified for the mock transfected cells as expected (lane 5, 6). These results demonstrated that the constructed JEV replicon could efficiently replicate and express nonstructural protein in transfected cells.

**Figure 2:**
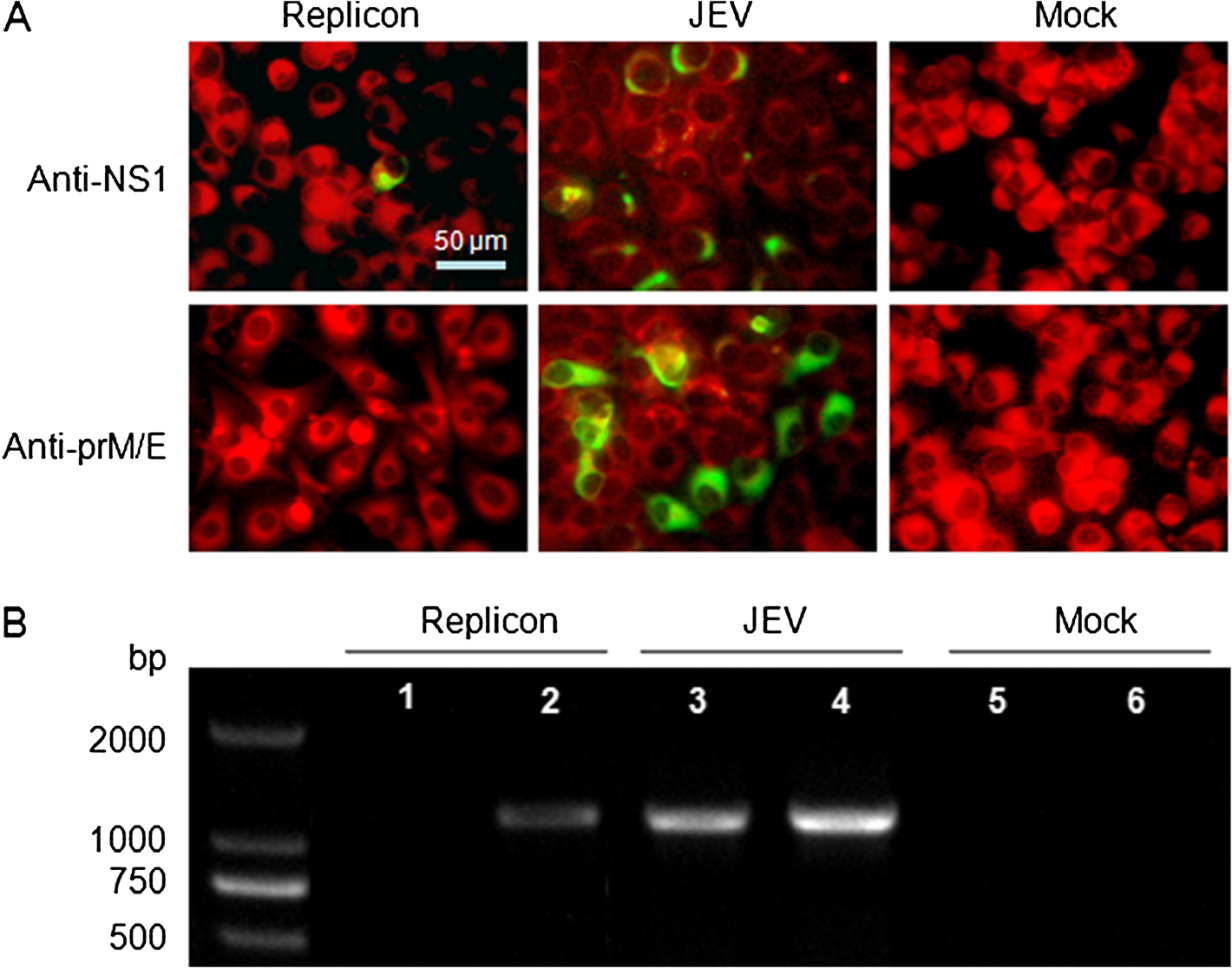
**Characterization of the JEV replicon.** (**A**) BHK-21 cells transfected with the JEV replicon pJE3Rep were subjected to indirect immunofluorescence assay (IFA). JEV viral proteins were visualized using NS1- and E-specific mouse monoclonal antibody and stained with fluorescein isothiocyanate (FITC)-conjugated goat anti-mouse IgG at 72 h post-transfection. The green signals represent FITC-positive cells. Viral RNAs (JEV) and PBS (Mock) were set as controls. (**B**) RT-PCR targeted at the prM/E genes (Lane 1, 3 and 5) and NS3 genes (Lane 2, 4 and 6). The amplified products for NS3 and prM/E gene were 1185 and 1208 bp, respectively. Total RNAs were extracted from BHK-21 cells at 72 h post transfection. Viral RNAs (JEV) and PBS (Mock) were set as controls.

To further adapt the potential application of JEV replicon system, a series of JEV replicons expressing the enhanced green fluorescent protein (EGFP) reporter gene (Figure 1) were constructed. A DNA fragment encoding 2A protease of foot-and-mouth disease virus (FMDV-2A) in fusion with the downstream region of the EGFP encoding sequence was amplified from pEGFP-N1 vector (Promega) using primer set F-KAS-EGFP and J-2A-E3-R. The resulting fusion PCR fragment was digested by *Kas*I and *Bsp*EI and ligated into the pJE3Rep, yielding the JEV reporter-replicon pJ/EGFP2A/E3Rep. Another two JEV reporter-replicons retaining the C-terminal 25 and 71 amino acids residues of E protein, pJ/EGFP2A/E25Rep and pJ/EGFP2A/E71Rep, respectively, were constructed. All the molecular constructs were prepared by using standard molecular biology techniques, and confirmed by restriction digest analysis and DNA sequencing.

To measure the expression of EGFP reporter gene, equal amounts of RNA transcripts derived from pJ/EGFP2A/E3Rep, pJ/EGFP2A/E25Rep and pJ/EGFP2A/E71Rep were transfected into BHK-21 cells. Transient expression of EGFP in transfected cells was visualized under light and fluorescence microscopy at 48, 72, 96 and 120 hours post transfection. As shown in Figure 3, EGFP-positive cells were observed in all the replicon-transfected BHK-21 cells, indicating that the reporter gene was successfully expressed in the cells. An increase of EGFP-positive cells could be observed in the early time post transfection, indicating the success of self-replication of the JEV replicon. Then, the EGFP-positive cells began to reduce at about 120 h post transfection. The EGFP-positive rate in the pJ/EGFP2A/E3Rep-transfected cells was relatively higher than that in pJ/EGFP2A/E25Rep- and pJ/EGFP2A/E71Rep-transfected cells. And the pJ/EGFP2A/E71Rep replicon showed the lowest positive rate among the three JEV replicons. These results indicated that the remaining amino acid length at the C-terminal of E protein could potentially affect JEV replication. Additionally, no obvious cytotoxicity was observed for all three replicons under light microscope, which is in agreement with previous results from YFV replicons [19]. Thus, all these JEV EGFP reporter-replicons could replicate efficiently and express EGFP proteins in BHK-21 cells.

**Figure 3:**
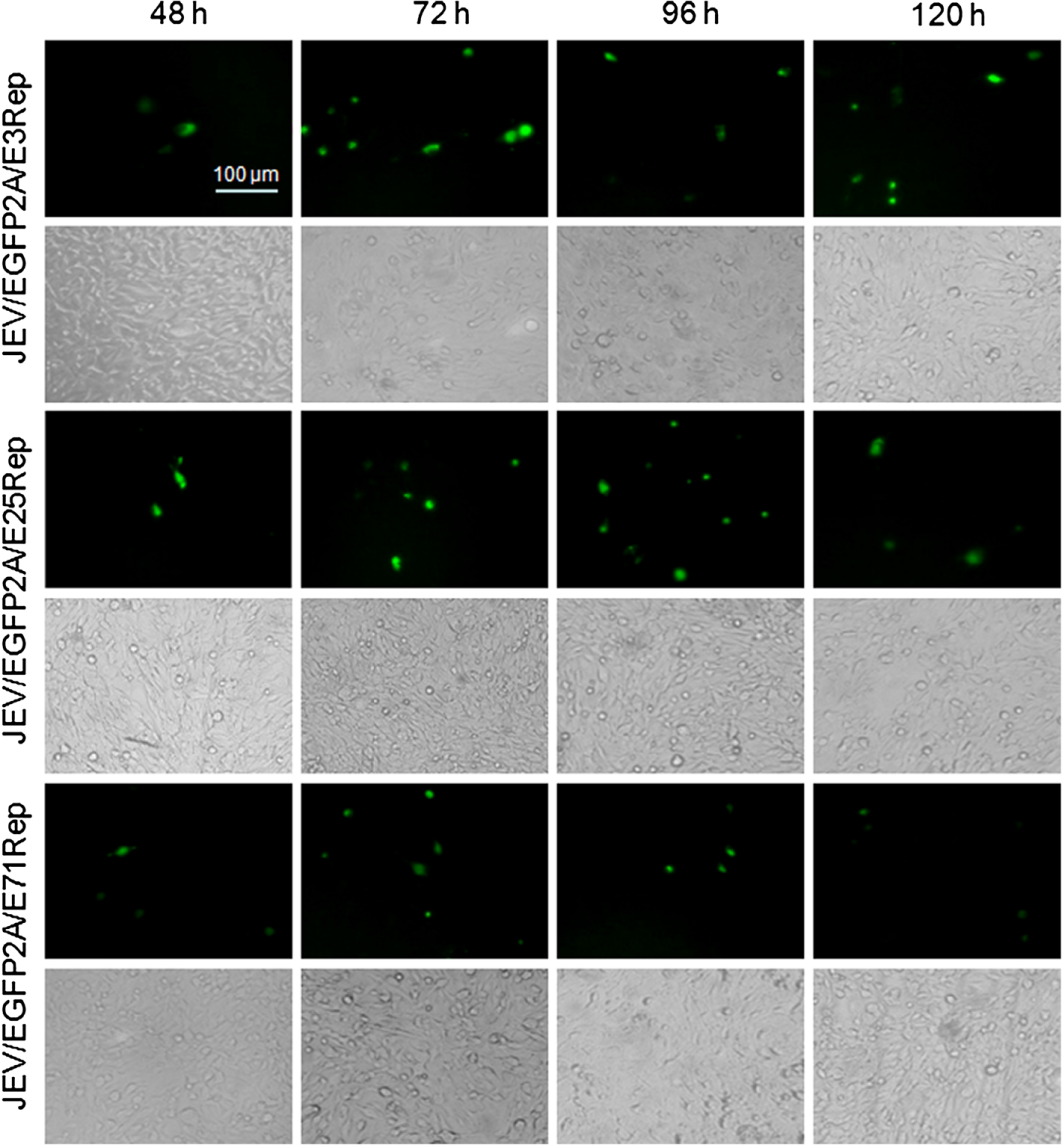
**Characterization of the JEV EGFP reporter replicons.** BHK-21 cells were transfected with RNA transcripts from linearized pJ/GFP/E3Rep, pJ/GFP/E25Rep and pJ/GFP/E71Rep, respectively and the expression of EGFP was observed under light and fluorescence microscopy at the indicated time points post transfection.

Finally, the Renilla luciferase (R.luc) reporter JEV replicon, pJ/R.luc2A/E3Rep, was constructed using the same strategy as the EGFP replicon (Figure 1) and the R.luc gene was amplified from pRL-CMV vector (Promega). To observe the expression of R.luc, *in vitro* RNA transcripts of pJ/R.luc/E3Rep were transfected into BHK-21 cells as previously described. For luciferase assay, the transfected cells were lysed in 100 μl of passive lysis buffer (Promega) per well of a 48-well plate at 24, 48, 72, 96, 120 and 144 h post transfection, respectively. Triplicate wells were seeded for each time point. In general, luciferase activity assays were initiated by mixing 20 μl of prepared cell extract with 100 μl of the appropriate R.luc substrate, and then measured by using a Glomax system (Promega) according to the manufacturer’s instruction. The results showed that the R.luc activity firstly peaked at 24 h post transfection, then declined rapidly from the first peak. After that, the R.luc activity began to rise stably until it reached a second peak at 144 h post transfection (Figure 4). Luciferase activity at early time points is indicative of translation of the input replicon RNAs, while the second peak at later time points represents proteins expressed by newly synthesized RNAs [33]. Additionally, IFA and RT-PCR assays also confirmed that the pJ/R.luc2A/E3Rep efficiently expressed the NS1 proteins and synthesized viral RNA of JEV at 72 h post transfection (data not shown). These results indicated that this R.luc reporter JEV replicon could replicate efficiently and express R.luc proteins in BHK-21 cells.

**Figure 4:**
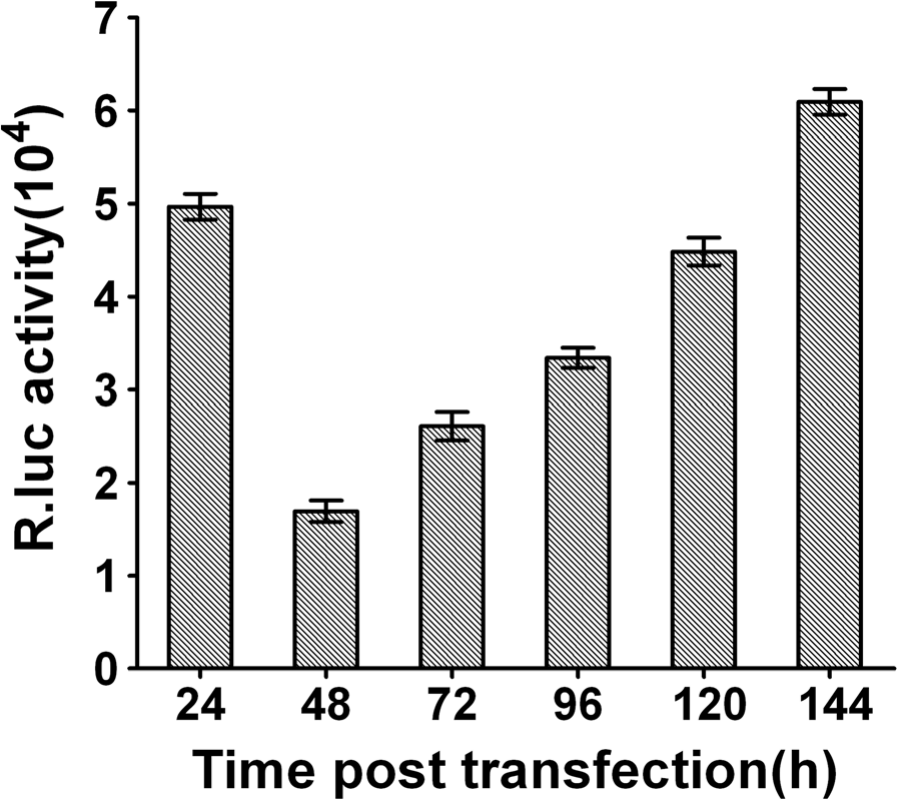
**Characterization of the JEV R.Luc reporter replicon.***In vitro* RNA transcripts of pJ/R.luc/E3Rep were transfected into BHK-21 cells, and the cells were harvested at the indicated times post transfection, the luciferase activity was measured by using a Glomax system (Promega) according to the manufacturer’s instruction. The results represent the means and standard deviations derived from three independent experiments performed in triplicate.

Previously, we have generated the infectious clone of JEV and constructed mutant JEV by using reverse genetic technology [34]. In this study, we have constructed a series of JEV replicons based on the JEV live vaccine strain SA14-14-2, and all these replicons are functionally active to replicate and express the desired foreign reporter genes. These replicons constructed herein are under the control of SP6 promoter. Previously, some DNA-based JEV replicons that under the control of CMV promoter have been developed and adapted for pseudo infectious particles [35,36]. These JEV replicons not only help to understand the molecular mechanism of viral replication, but also provide a powerful tool for foreign proteins expression, chimeric vaccine and single-round virus like particles (VLP) based vaccine development. The live vaccine virus SA14-14-2 has been widely used in the most JEV endemic countries owing to its highly efficiency, and very few adverse effects [37-39]. Another flavivirus live vaccine strain, YFV 17D, has been widely used as genetic backbone for chimeric flavivirus vaccine development [40-45]. The potential applications of JEV SA14-14-2 in vaccine development are of high significance and deserve further investigation [11,46,47]. Currently, we are working with these JEV replicons to generate a series of chimeric flaviviruses vaccine candidates.

## Competing interests

The authors declare that they have no competing interests.

## Authors’ contributions

SHL and XFL carried out most of the experiments and drafted the manuscript. HZ, YQD, XDY, SHZ, JT, QY, EDQ participated in experiments and data analysis. CFQ designed the study, supervised the work and edited the final version of this manuscript. All authors have read and approved the final version of the manuscript.

## Supplementary Material

Additional file 1: Table S1 Oligonucleotide primers for construction of JEV replicons and inserting reporter genes. Click here for file
