# Supplementary material for: Development and characterization of the replicon system of Japanese encephalitis live vaccine virus SA14-14-2
Source: Virol J. 2013 Feb 26;10:64. doi: 10.1186/1743-422X-10-64 (PMC3608946; doi:10.1186/1743-422X-10-64)
Supplement: Additional file 1: Table S1 — Oligonucleotide primers for construction of JEV replicons and inserting reporter genes. [file 1743-422X-10-64-S1.docx]

**Table S1. Oligonucleotide primers for construction of JEV replicons and inserting reporter genes**

| Primer^a^ | Sequence (5’ to 3’) ^b^ | Position^c^ | | Restriction sites |
| --- | --- | --- | --- | --- |
| Not I（+）  linker-1（-）  pBRJEV-7289(+)  pBRJEV-7289(-)  pBRJEV-7289-3U(+)  pBRJEV-7289-3U(-)  R-J-BglⅡ  J-2A-E25-F  J- 2A-E25-R  J- 2A-E71-F  J -2A-E71-R  F-KAS-EGFP  F-KAS-R.luc  J-2A-E3-R  J-2A-E3-F  J-BspEI(-)  F-KAS-JEV  F-Asc I  F-J-Rep  R-J-Rep  R13  F2  R2  F6  R6 | CGAC**GCGGCCGC**GCTAGCGATGAC  GTCCTCGAGTCCGGAGGCGCGCCCGTATGTGTATGATACATAAGG  GGCGCGCCGACACATGGAAACTCGAGAGGGCAGT  GCCATCCCGGGAGCATGTA  GGCGCGCCCTCGAGGGTACATGCTCCCGGGAT  CTCGAGAGATCCTGTGTTCTTCCTCAC  GTGAC**AGATCT**GACTCCGCACACGC  CGTCGAGTCCAACCCTGGCCCCCGAGACCGATCAATTGCT  AGCAATTGATCGGTCTCGGGGGCCAGGGTTGGACTCGACG  CGTCGAGTCCAACCCTGGCCCCATTGGAGGGGTCTTCAACTC  GAGTTGAAGACCCCTCCAATGGGGCCAGGGTTGGACTCGACG  TGTGCA**GGCGCC**ATGGTGAGCAAGGGCGAGGAGCTGTTCACCGGGGTGGTG  TGTGCA**GGCGCC**ATGACTTCGAAAGTTTATGATCCAGAACAAAGGAAACGG  CATCCAGTGTCAGCATGCACGGGGCCAGGGTTGGACTCGACG  CGTCGAGTCCAACCCTGGCCCCGTGCATGCTGACACTGGATG  CCGTACCAGCAGCCATTTTCTG**TCCGGA**ATCGTAGG  GCTTGTGCA**GGCGCC**ATGAAGTTGTCG  GCTG**GCGCGC**CATTTAGGTGACACTATAGAGAAGTTTATCTGTGTGAAC  GTCATAGCTTGTGCA**GGCGCC**GTGCATGCTGACACTGGATG  GTGAC**AGATCT**GACTCCGCACACGC  AGATCCTGTGTTCTTCCTCACCACCAGCTACA  AGAGGCTTGGCTGGATTCAA  TCGCAACGGAAACAATCGGA  GGGATGAGGGCTCTATACCT  ACCAGGGTGCAAATCTAGCA | | 3099-3124  7281-7299  7279-7296  10956-10976  2637-2661  2403-2420  2403-2420  2265-2284  2265-2284  465-476  465-476  2469-2488  2469-2488  3437-3472  462-488  2469-2488  2635-2659  10945-10976  800-819  1988-2007  4014-4013  5179-5198 | *Not* I  *Bgl*Ⅱ  *Kas* I  *Kas* I  B*spE*I  *Kas* I  *Asc* I  *Kas* I  *Bgl*Ⅱ |

^a^ F, viral genomic sense; R, complementary sense.

^b^Viral sequences are underlined. Restriction sites are bold.

^c^Number represents base position in the genome of JEV strain SA14-14-2 (GenBank access number: D90195).
